# Translation and cross-cultural adaptation of the Quality of Palliative Care in the Intensive Care Unit questionnaire

**DOI:** 10.62675/2965-2774.20250345

**Published:** 2025-07-15

**Authors:** Eduardo Tavares Gomes, Tânia Couto Machado Chianca

**Affiliations:** 1 Universidade Federal de Minas Gerais Escola de Enfermagem Belo Horizonte MG Brazil Escola de Enfermagem, Universidade Federal de Minas Gerais - Belo Horizonte (MG), Brazil.

## INTRODUCTION

The discussion of palliative care (PC) in intensive care units (ICUs) is urgent and becomes even more relevant when we consider that up to 20% of deaths occur during an ICU stay or shortly after discharge, indicating the need for the preparation and support of intensivist professionals to address the process of death and dying.^(1,2)^

Today et al., in a study that aimed to evaluate and compare the quality of PC in ICUs, developed and validated a questionnaire for this purpose.^(3,4)^

The questionnaire, which is composed of 10 Likert-type questions, assesses seven PC domains: communication within the team, decision-making with the patient and family, continuity of care, emotional and practical support, symptom management, and spiritual and emotional support for health professionals. The final score is the average of the scores assigned to each item, ranging from zero (lowest quality) to 10 (highest quality).^(3,4)^

Thus, the present study aimed to translate and cross-culturally adapt the Quality of Palliative Care in the Intensive Care Unit questionnaire into Brazilian Portuguese.

## METHODS

This study was conducted between July and September 2024 and followed steps adapted from Beaton et al. and the document Best Practices for Developing and Validating Scales for Health, Social, and Behavioral Research,^(5,6)^ as described below:

1. Initial translation: this translation was performed by three Brazilian translators, all proficient in English, one health professional with a doctorate and extensive experience in international publications in the field of intensive care.
2. Translation synthesis: the versions generated by the three translators were gathered and synthesized into a single preliminary version, with the goal of identifying and resolving discrepancies. One of the researchers, who has a PhD, command of the English language and expertise in the field, conducted this synthesis, which ensured methodological rigor in preserving the original meaning of the instrument.
3. Back-translation: the consensus version was translated back into English by two independent translators, with one translator being an American and the other a Brazilian naturalized American residing outside Brazil (United States); both have command of the Portuguese language and Brazilian culture. One of the translators was a health professional who is currently retired. The goal of this step was to ensure that the semantic content was maintained.
4. Review by a Committee of Experts: the expert committee consisted of two physicians and five nurses (all with at least two years of experience in intensive care and at least a master's degree) and two linguists. One meeting was sufficient for discussing semantic, idiomatic, experiential (relevance to the target population) and conceptual equivalences and assessing whether the terms and concepts had similar meanings in different languages and cultures.^(6)^
5. Pretest: in the last stage, specialists in nursing and medicine, with at least two years of experience in intensive care and training in the field, were invited to participate in the validation of the instrument. The invitations were sent by email along with the Free and Informed Consent Form, as well as evaluation guidelines and a link to fill out the form on the Redcap platform. For this stage, the initial goal was to evaluate more than 25 experts, according to the reference used, which recommends 30 to 40 evaluators.^(5,6)^ In total, 42 invitations were sent, and 25 responses were obtained, 17 from nurses and eight from physicians.

To evaluate content validity, the content validity index (CVI) and the kappa coefficient were calculated, considering a minimum acceptable value of 0.75.^(5,6)^ The CVI calculation was based on the proportion of experts who rated the item as 3 or 4 on the relevance scale, dividing this number by the total number of respondents, resulting in a score between 0 and 1.00.

Authorization from the authors of the original instrument had previously been requested. Approval of the Research Ethics Committee was obtained under protocol number 7,081,789.

## RESULTS

Table 1 presents the preliminary version after the translations and the final version were completed after the meeting with the judges. The Committee of Judges also discussed and reached a consensus on the abbreviation to be used for the title of the *Qualidade dos Cuidados Paliativos na Unidade de Terapia Intensiva* in Portuguese, defined as the QCP-UTI.

**Tabla 1:** Preliminary consensus versions and final versions of the *Qualidade dos Cuidados Paliativos na Unidade de Terapia Intensiva* in Portuguese

*Versão consensual preliminar*	*Versão final em português*
*Título:* *Questionário: Qualidade dos cuidados paliativos na unidade de terapia inte* *nsiva – QCP-UTI*	*Título:* *Questionário: Qualidade dos cuidados paliativos na unidade de terapia inte* *nsiva - QCP-UTI*
*Subtítulo:* *Avaliação geral da qualidade dos cuidados paliativos prestados por médicos e enfermeiros na sua UTI*	*Subtítulo:* *Avaliação geral da qualidade dos cuidados paliativos prestados por médicos e enfermeiros na sua UTI*
*Instruções:* *Pedimos que você dê respostas pontuando de 0 a 10 em cada item sobre a qualidade geral das práticas de cuidados paliativos de médicos e de enfermagem. Perguntamos aqui: Até que ponto os médicos e enfermeiros da sua UTI prestam cuidados paliativos em cada um dos sete domínios de cuidados de fim de vida? Por favor, escolha uma resposta para todas as perguntas.*	*Instruções:* *Pedimos que você dê respostas pontuando de 0 a 10 em cada item sobre a qualidade geral das práticas de cuidados paliativos prestados por médicos e enfermeiros na sua UTI. Perguntamos aqui: Até que ponto os médicos e enfermeiros da sua UTI prestam cuidados paliativos em cada um dos sete domínios de cuidados de fim de vida? Por favor, escolha uma resposta para todas as perguntas*
*Domínio 1: Comunicação Dentro da Equipe e com Pacientes e Familiares*	*Domínio 1: Comunicação Dentro da Equipe e com Pacientes e Familiares*
*Item 1. Comunicação com membros da equipe clínica para esclarecimento dos objetivos do cuidado*	*Item 1. Comunicação com membros da equipe assistencial para esclarecimento dos objetivos do cuidado*
*Item 2. Comunicação com pacientes e familiares sobre objetivos de cuidado e tratamento*	*Item 2. Comunicação com pacientes e familiares sobre as metas de cuidado e tratamento*
*Domínio 2: Decisão Centrada no Paciente e na Família*	*Domínio 2: Tomada de decisão Centrada no Paciente e na Família*
*Item 3. Elicitar e respeitar as preferências do paciente e/ou família em relação aos objetivos de cuidado e tratamento*	*Item 3. Investigar e respeitar as preferências do paciente e/ou família em relação às metas de cuidado e tratamento*
*Domínio 3: Continuidade do Cuidado*	*Domínio 3: Continuidade do Cuidado*
*Item 4. Comunicação com colegas sobre as necessidades emocionais do paciente e/ou família*	*Item 4. Comunicação com colegas da equipe assistencial sobre as necessidades emocionais do paciente e/ou família*
*Item 5. Comunicação dos objetivos do cuidado aos próximos cuidadores*	*Item 5. Comunicação das metas de cuidado aos próximos profissionais (médico ou enfermeiro)*
*Domínio 4: Apoio Emocional e Prático para Pacientes e Familiares*	*Domínio 4: Apoio Emocional e Prático para Pacientes e Familiares*
*Item 6. Atenção às necessidades emocionais e práticas dos pacientes terminais e dos seus familiares*	*Item 6. Atenção às necessidades emocionais e práticas dos pacientes em estágio terminal de doença e dos seus familiares*
*Domínio 5: Gestão de Sintomas e Cuidados de Conforto*	*Domínio 5: Gestão de Sintomas e Cuidados de Conforto*
*Item 7. Gestão dos sintomas e prestação de cuidados de conforto*	*Item 7. Gestão dos sintomas e garantia de cuidados de conforto*
*Domínio 6: Apoio Espiritual para Pacientes e Familiares*	*Domínio 6: Apoio Espiritual para Pacientes e Familiares*
*Item 8. Avaliação das necessidades espirituais/religiosas do paciente e família*	*Item 8. Avaliação das necessidades espirituais/religiosas do paciente e família*
*Domínio 7: Apoio Emocional e Organizacional para Médicos de UTI*	*Domínio 7: Apoio Emocional e Organizacional para Médicos de UTI*
*Item 9. Fornecimento de apoio emocional para médicos que cuidam de pacientes terminais*	*Item 9. Fornecimento de apoio emocional para profissionais que cuidam de pacientes em estágio terminal de doença*
*Item 10. Oferta de educação sobre cuidados paliativos*	*Item 10. Oferta de educação sobre cuidados paliativos (para profissionais)*
*Conclusão:* *Obrigado por dedicar seu tempo para preencher esta pesquisa. Sabemos que o seu tempo é importante e que há muitas demandas para você. A sua contribuição é essencial para os esforços contínuos para melhorar os cuidados paliativos na UTI*.	*Conclusão:* *Obrigado por dedicar seu tempo para preencher esta pesquisa. Sabemos que o seu tempo é importante e que há muitas demandas para você. A sua contribuição é essencial para os esforços contínuos para melhorar os cuidados paliativos na UTI*.

*UTI - unidade de terapia intensiva.*

The CVI was used to assess the relevance and clarity of each item, whereas the kappa coefficient was applied to measure the level of agreement among experts on the properties of the instrument (Table 2).

**Table 2:** Results of the construct validity index and the kappa coefficient for the ten items of the Quality of Palliative Care in the Intensive Care Unit (N=25)

Item	Equivalences						Clarity		Relevance	
	Semantics		Conceptual		Cultural					
	CVI	Kappa	CVI	Kappa	CVI	Kappa	CVI	Kappa	CVI	Kappa
1	0.98	0.98	0.93	0.93	0.92	0.92	0.91	0.91	0.98	0.98
2	0.96	0.96	0.96	0.96	0.92	0.92	0.92	0.92	0.98	0.98
3	0.94	0.94	0.91	0.91	0.90	0.90	0.92	0.92	0,94	0.94
4	0.93	0.93	0.93	0.93	0.91	0.91	0.81	0.81	0.93	0.93
5	0.94	0.94	0.92	0.92	0.93	0.93	0.91	0.91	0.98	0.98
6	0.94	0.94	0.94	0.94	0.96	0.96	0.83	0.83	0.97	0.97
7	0.91	0.91	0.89	0.89	0.87	0.87	0.91	0.91	0.93	0.93
8	0.93	0.93	0.93	0.93	0.91	0.91	0.92	0.92	0.89	0.89
9	0.94	0.94	0.92	0.92	0.92	0.92	0.79	0.79	0.89	0.89
10	0.89	0.89	0.89	0.89	0.86	0.86	0.78	0.78	0.90	0.90

CVI - construct validity index.

The results indicate good content validity, with all the items presenting indices greater than 0.79, which is considered satisfactory according to the established criteria (Table 2). These results demonstrate that the items of the ICU-QCP questionnaire have semantic, conceptual and cultural adequacy for the Brazilian context and are potentially valid for the evaluation of the quality of PC in Brazilian ICUs.

## DISCUSSION

The ICU-QCP items address domains widely recognized in the literature as essential for the implementation of effective PC.^(1–4,7–11)^ The high relevance scores assigned by the experts to all ten items confirm the importance of aspects such as communication and the inclusion of patients and family members in decision-making, standing out as fundamental elements for the provision of patient-centered care.

In general, all ICU-QCP items were considered valid for use in Brazil, with a high level of agreement among experts. Items 9 and 10 presented results slightly below the other items in terms of clarity but within acceptable limits. In the evaluation phase of the psychometric properties, it is necessary to evaluate whether there is a need to revise the wording of these or if the performance of the items in the scale is adequate.

In another study with nurses from Israel, the authors reported moderate results in the perception of professionals about PC in their ICUs; however, the lowest scores were reported for Items 9 and 10.^(4)^ These items should be the subject of future revisions to improve their cultural equivalence and clarity. In another study conducted with Chinese intensivist professionals, items from another instrument also had lower scores in the evaluation of PC for items about the support offered to professionals (the same evaluation area as Items 9 and 10 of the ICU-QCP).^(11)^

This instrument is relevant because it provides a form of evaluation that is easy to apply in ICUs, with great potential for evaluating interventions in the field of PC in ICUs. The QCP-UTI allows the analysis of PC areas and indicates weaknesses for possible interventions that will improve the quality of services provided.

A limitation of this study was the absence of translators specializing in PC or intensive care. However, the Committee of Judges made technical adjustments that adapted the terms used to the specific area, ensuring the necessary precision.

## CONCLUSION

After the review stage by the Committee of Judges, the final version of the instrument *Qualidade dos Cuidados Paliativos na Unidade de Terapia Intensiva* did not undergo further changes, and high results in the indices of content validity and the kappa coefficient were obtained. The instrument was translated and culturally adapted with good content validity and is ready for future studies to evaluate its psychometric properties.
